# Two‑year clinical performance of indirect restorations fabricated from CAD/CAM nano hybrid composite versus lithium disilicate in mutilated vital teeth. A randomized controlled trial

**DOI:** 10.1186/s12903-023-03847-6

**Published:** 2024-01-17

**Authors:** Haneen Ahmad Shafik Elmoselhy, Olfat EL Sayed Hassanien, Mohamed Fouad Haridy, Maha Abd El Salam El Baz, Shehabeldin Saber

**Affiliations:** 1 Conservative Dentistry Department, Faculty of Dentistry, The British University in Egypt, Suez Desert Road El Sherouk City, Egypt; 2 Conservative Dentistry Department, Faculty of Dentistry, Cairo University, Giza, Egypt; 3 Conservative Dentistry, Faculty of Dentistry, Cairo University and The British University in Egypt, Suez Desert Road El Sherouk City, Egypt; 4 Endodontics, Faculty of Dentistry, Ain Shams University and The British University in Egypt, Suez Desert Road El Sherouk City, Egypt

**Keywords:** Randomized controlled trial, Dental porcelain, Lithium disilicate: CAD/CAM, Nano-hybrid composite, Inlays

## Abstract

**Trial design:**

This is a randomized, controlled, superiority, double-blinded, parallel-group, two-arms trial with an allocation ratio of 1:1. The aim of this trial was to compare the two-year clinical performance of partial indirect restorations fabricated from CAD/CAM nano-hybrid composite and ceramic lithium disilicate blocks using the modified USPHS criteria.

**Methods:**

In two parallel groups (*n* = 50 restorations), fifty participants having mutilated vital teeth with a minimum of two remaining walls were randomly enrolled in this trial and received indirect restorations of either nano-hybrid composite resin blocks (Brilliant, Coltene, Switzerland) or Lithium Disilicate (IPS Emax CAD). The restorations were assessed using modified USPHS criteria by two independent blinded assessors at baseline, six months, one-year and two years follow-up visits. Categorical and ordinal data were presented as frequencies and percentages. Categorical data were analyzed using the chi-square test. Ordinal data were analyzed using the Mann-Whitney U test for intergroup comparisons and Freidman’s test followed by the Nemenyi post hoc test for intragroup comparisons. Numerical data were presented as mean and standard deviation values. They were analyzed for normality using the Shapiro-Wilk test. Data were found to be normally distributed and were analyzed using the independent t-test. The significance level was set at *p* ≤ 0.05 within all tests.

**Results:**

Forty-eight participants received the allocated intervention and completed the follow-up periods. There was a statistically significant difference between both tested materials for all USPHS criteria regarding Marginal integrity and Marginal discoloration at six-months Follow-up, but with no statistically significant difference at one- and two-year follow-up.

**Conclusions:**

Both materials showed an acceptable, successful clinical performance along the two-years follow-up period.

**Clinical relevance:**

The CAD/CAM nano-hybrid composite blocks are as reliable as Lithium disilicate for restoring mutilated vital teeth.

## Introduction

The choice of the most appropriate technique and material to restore large cavities with weakened cusps still creates doubts among clinicians [1]. To obtain an ideal anatomy, contour and contact, indirect partial coverage restorations are favored [2]. Although they offer adequate resistance to fracture and wear [3], clinical fracture is not uncommon [4].

Recently, there has been an increasing interest in fabricating such restorations using the CAD/CAM technology. This offers the opportunity to scan, design, and fabricate the restoration in a single appointment without making impressions, provisional restorations, or dental laboratory support. Moreover, industrially fabricated blocks are more homogeneous, and have higher intrinsic strength than the materials used for direct restorations [4].

Different ceramic systems are available for constructing restorations using CAD/CAM technology. This includes blocks made with glass ceramics, resin nano ceramics, zirconia, ceramic composites, ceramics and resin composites [5]. IPS e.max CAD (Ivoclar-Vivadent, Liechtenstein, Germany) is a milling lithium disilicate-reinforced ceramic with a high crystalline content of up to 70 vol% in a glass matrix. It comes as a pre-crystallized block, containing 32 vol% of metasilicate (Li_2_SiO_3_) crystals and 0.7 vol% lithium disilicate (Li_2_Si_2_O_5_) crystal nuclei, displaying a flexural strength of around 130 MPa, making the milling process easier. It is later crystallized in a ceramic oven at 850 °C in a vacuum for 20–25 min, where the metasilicate is dissolved and crystallizes as lithium disilicate changing from a bluish color to the chosen shade and translucency and increasing its flexural strength to around 360 MPa [6, 7].

CAD/CAM composites have superior mechanical properties to direct resin composites [8–10] due to the innovative composition and polymerisation modes under high temperature and pressure [11]. This category of materials merges the favourable properties of ceramics such as durability, enamel-like surface finish, good esthetics, and colour stability, with the favourable properties of composite resin, such as high flexural strength, low abrasiveness, and ease of polishing [12, 13]. Another possible advantage of resilient ceramic materials is that the adhesive resin cements may have a more similar wear rate than the restoration, leading to improved marginal integrity over time [14]. Brilliant Crios (Coltène Whaledent, Switzerland) is a nano-hybrid composite that contains barium glass (< 1.0 μm), amorphous silica (< 20 nm) with a filler wt of 70.7% in addition o resin matrix cross-linked methacrylates and inorganic pigments such as ferrous oxide or titanium dioxide.

Currently, scarce clinical data exists on the long-term outcomes of posterior partial indirect restorations fabricated from either CAD ceramic or composite blocks [15, 16]. Therefore, this study sought to evaluate the clinical performance of restorations fabricated from either IPS e.max CAD or Brilliant Crios nano-hybrid composite blocks regarding marginal integrity, marginal discoloration and restoration fracture using the modified USPHS criteria. The null hypothesis tested is that both materials will present a similar clinical performance after two years of clinical use.

## Materials and methods

All materials and their description, composition, lot number and manufacturer are listed in Table 1.

**Table 1:** Technical information of the materials used in the clinical study

Material	Description	Composition	Lot number	Manufacturer
**BRILLIANT Crios**	Nano-hybrid composite blocks	barium glass (size < 1.0 μm), amorphous silica (size < 20 nm), resin matrix cross-linked methacrylates and inorganic pigments such as ferrous oxide or titanium dioxide	J46379	Coltène Whaledent, Switzerland
**IPS E.max CAD**	Lithium disilicate blocks Shade A3.5 HT	SiO_2_ (57–80%), Li_2_O (11–19%), K_2_O (0–13%), P_2_O_5_ (0–11%), ZrO_2_ (0–8%), Al_2_O_3_ (0–5%), MgO 90–5%) and coloring oxides (0–8%)	W82636	Ivoclar Vivadent, Liechtenstein, Germany
**Rubber Dam**	Dental Dam	Powder-free Latex rubber dam sheets	DD01HKS1	Sanctuary, Malaysia
**IPS Ceramic Etching Gel**	4% buffered HF acid gel			Ivoclar Vivadent, Liechtenstein, Germany
**Calibra silane**	One-component silanization agent	Phosphoric acid ester, trimethoxysilane and acetone	1,647,247	Dentsply Sirona, Milford, USA
**Acid etchant**	Phosphoric acid etchant	Water, 37%Phosphoric acid, Synthetic amorphous silica, Polyethylene glycol, Aluminum oxide	J50811	Coltène Whaledent, Switzerland
**Brilliant EverGlow™** **Flow**	Bulk fill flowable composite	TEGDMA – BISGMA – Ytterbium Trifluoride – Zinc Oxide – Dental glass – Amorphous silica. Filler particles: 0.02–1.5 μm Inorganic filler content by volume: 37% Inorganic filler content by weight: 60%	1,806,263	Coltène Whaledent, Switzerland
**Duocem**	Dual-cured adhesive resin cement	Bis-GMA, DMA, silica fillers, benzoyl peroxide, amines, pigments, additives	I71056	Coltène Whaledent, Switzerland
**One coat 7 active universal adhesive**	Universal adhesive (mild pH > 2.5)	Urethane dimethacrylate 2-hydroxyethyl methacrylate Photoinitiators, Ethanol, Ethyl alcohol, Water	J35422	Coltene, Switzerland
**Aluminium Oxide**	Abrasive powder	Oxide Aluminium 53 μm particle size Velopex international, UK	100,119	Velopex international, UK
		Oxide Aluminium 29 μm particle size Velopex international, UK		

### Study setting

Approval of the study design was provided by the Research Ethics Committee at Cairo University, Egypt (CREC) (approval number: 16-09-2020, Date: 24-09-2020). Participants were recruited from the the Conservative Dentistry Department outpatient clinic, Faculty of Dentistry, Cairo University. All the patients were informed about the treatment’s indications, benefits, risks, and possible complications. All participants signed a written informed consent form. All procedures performed in this study were by with the Helsinki Declaration. The study protocol was registered in (www.clinicaltrials.gov) database, with unique identification number NCT04563624 on the date (24/09/2020).

### Trial design

The study design for this randomized controlled clinical trial was a double-blinded, parallel-group, two-arms, superiority trial with an allocation ratio of 1:1.

### Sample size calculation

A power analysis was designed to have adequate power to apply a statistical test of the null hypothesis that CAD/CAM indirect restorations fabricated from ceramic blocks are superior to those fabricated from composite blocks regarding their marginal adaptation after 24 months. According to the results of Fasbinder et al. [17] in which, the probability of score alpha-1 for marginal adaptation of CAD/CAM indirect ceramic restorations was (0.757), the probability of alpha-2 score was (0.243) with effect size w = 0.514 (*n* = 30). If the probability of score alpha-1 for marginal adaptation of indirect CAD/CAM composite restorations was (0.914), the probability of alpha-2 score was (0.086) with effect size w = 0.828 (*n* = 12), by adopting an alpha (α) level of 0.05 (5%), power = 80%. The predicted sample size was 42 (21 per group). The sample size was increased by (20%) to account for possible dropouts during follow-up intervals to be a total of [50] cases, i.e. [25]. for each group. Sample size calculation was performed using G*Power 3.1.9.2 using chi-square test with a superiority framework.

### Eligibility criteria

#### Inclusion criteria

Patients included in the study were healthy males and females (Category: American Society of Anesthesiologists class 1, aged 16–45 years, presenting with good oral hygiene, healthy periodontium, a single badly broken down vital molar (caries reaching > 1/2 of the dentin thickness on periapical digital radiographic examination, with at least two missing cavity walls and the cavo-surface margins in enamel), and the antagonist teeth present in normal occlusion.

#### Exclusion criteria

Patients presenting one of the following situations were not included in the study: the presence of systemic disease (ASA 2–6), pregnancy or breastfeeding, hypersensitive, endodontically treated, non-vital or cracked teeth, multiple teeth that required treatment, patients with wear facets and parafunctional habits as clinching and bruxism, allergy to the composite resin and adhesive system, high caries risk index, or active periodontal disease.

#### Randomization and blinding

Using computer-generated randomization (www.randomization.com), the participants who fulfilled the eligibility criteria were allocated randomly into two groups with a 1:1 allocation ratio (25 participants in each group). The sequentially generated numbers were placed by MH in opaque envelopes until the time of intervention. Participants were enrolled by OH. Each participant was asked to select an envelope that determined his/her group for future intervention. HA assigned participants to interventions. The participants and the statistician were blinded, while the operator could not be blinded due to the nature of the intervention used.

#### Intervention

Demographic data were recorded, and each patient’s medical and dental status and history were collected in charts. Clinical and radiographic examinations were performed, and their findings were registered.

### Restorative procedures

All operative procedures were performed with rubber dam isolation. All cavities were prepared according to the accepted principles for adhesive onlays [18]. A diamond stone # 245 (Komet, USA) and a straight fissure carbide bur number 57 size 010 (Komet, USA) were used to prepare the cavities. A new bur was used for every six preparations [19]. All carious dentin was excavated and removed by a hand excavator (#51/52, Maillefer Dentsply, Switzerland) according to the recent caries removal clinical recommendations [20]. Cuspal tipping for not less than 2 mm clearance, measured by a caliper (Diamonds Surgical Instruments, Pakistan), was done by a wheel stone (#909, Komet, USA) for weak cusps. Finishing was done using an extra-fine grit yellow-coded tapered with round-end diamond stones (#368EF, Komet, USA). The cavity walls were prepared with 12–15 degrees internal axial wall divergence using blue-coded diamond tapered with round end bur diameter 16, length 10 (MIDWEST Dentsply) held parallel to the long axis of the tooth.

During preparation, the following parameters checklist was followed for standardization [21]:

1. The thickness of the remaining walls, in order to maintain them, had to be ≥ 2 mm measured by a dental caliper.
2. The pulpal floor was prepared to provide a depth range of 3–4 mm measured by a periodontal probe.
3. The width of the occlusal isthmus had to be at least 2–3 mm.
4. Buccal and lingual walls of the proximal part of the cavity were prepared using the same diamond bur (Komet, USA) used for the occlusal part of the cavity to provide the same angle of divergence (12–15 degrees) as that of the occlusal walls.
5. The internal line angles were rounded, and the cavo-surface angles were 90°.
6. Regarding the cuspal coverage cases, the available inter-occlusal clearance was checked to be at least 2 mm in maximum intercuspation and during lateral movements.

### Cavity design optimization (CDO)

The cavity was optimized, if needed, using the flowable resin (Brilliant EverGlow™ Flow, coltene), which was placed to block existing internal cavity undercuts; followed by light-curing for 20 s using a LED light-curing device (Elipar™ Deep Cure, 3 M ESPE) of 1470 mw/cm^2^ light intensity. Post-curing through clear glycerin gel was done for an additional 20 s to minimize the formation of an oxygen inhibition layer. Before intra-oral scanning, proper cavity evaluation was done regarding the sharp margins, absence of undercuts or any sharp irregularities [22].

### Restoration construction

Each prepared tooth was scanned using the Omnicam intraoral camera of the CEREC system software version 4.60 (Sirona Dental Systems GmbH, D- 64,625 Benshein, Germany) for taking the optical impression [23]. Using the CEREC software version 4.60, the margin was drawn, and the final design was obtained and checked. The setting for the machining of composite restorations for occlusal and the lateral wall-thickness was entered to be 1.5 mm and with a 100 μm for the cement space. The MCXL milling machine (Sirona, USA) was used to mill the indirect restorations from nanohybrid CAD/CAM composite blocks (Brilliant blocs) size 14.

### Cementation procedures

Before the cementation procedure, proper cleaning of the sealed cavity was achieved with soft-air abrasion airborne particle abrasion with aluminium oxide after try-in of the restoration and before cementation [24]. The restoration fitting surface was cleaned and roughened with 50 microns aluminium oxide using an intraoral sandblaster unit (Aquacare, Velopex, UK) [25]. The restoration was then placed in an ultrasonic cleaner filled with distilled water for 4 min [26]. After removal from the cleaner, the fitting surface was gently air-dried. The adhesive was actively rubbed for 20 s (One coat7), and solvent evaporation was allowed for 20 s before light-curing for 10 s, according to the manufacturer’s instruction [27]. The tooth surface was activated for bonding by air abrasion with 29 microns aluminium oxide. Then, 37% phosphoric acid gel (Gel S, coltene, Switzerland) was first applied to the enamel margins for 15 s and for an extra 10 s on the cavity interiors, rinsed for 30 s and gently air dried. The universal adhesive One coat7 (coltene,Switzerland) was actively applied for 20 s, gently air-dried for 5 s, and light cured for 10 s. A dual-cured adhesive resin cement (Duocem,Coltene) was injected into the cavity using the auto-mix tip supplied by the manufacturer. The restoration was then placed in the cavity and checked for complete seating with an ultrasonic seating tip (G22, NSK, Japan). The cement was tac light-cured for 2 s to facilitate removal of the interproximal and marginal excess with dental floss, and then light curing was done from all directions each for 40 s, to achieve the final set.

### Contact, occlusal checking and finishing and polishing

The proximal contacts were checked with an unwaxed dental floss (Oral-B, USA). The occlusal contacts were adjusted using an articulating paper (Blue Red Combo 0.0028”/71 µm, Crosstex ® International, USA). Finally, finishing was done using fine grit yellow coded tapered with round and flame diamond stones (#368EF, #852EF, Komet, USA) while polishing was done by rubber points (Enhance kit, Dentsply Sirona) operated at low-speed contra-angle handpiece (NAC-EC, NSK, Japan) with a maximum speed 20,000 rpm under water coolant and minimal pressure.

### Lithium disilicate indirect restorations

All the steps were repeated as discussed before, except for crystallization and cementation. After milling, the blue un-crystallized milled restorations were trimmed carefully using diamond abrasives at a very low speed to remove excess material at the connection site with the ceramic block, and was checked intra-orally to ensure complete seating of the restoration. The restoration margins were checked along with the proximal and occlusal contacts. The restorations were then introduced into the furnace (CEREC SpeedFire, Dentsply Sirona, Milford, USA). For the crystallization cycle according to the preset firing parameters. After adjustments and cooling of the restorations, they were checked intraorally. The glazing cycle was done according to the preset glazing parameters instructed by the manufacturer [23]. Before cementation, the restoration fitting surface was treated with 4% hydrofluoric acid for 60 s and rinsed with water according to the manufacturer’s instructions. The surface was cleaned with 37% phosphoric acid gel (Gel S)) for 60 s, rinsed and gently dried by the air stream. The Cleaned restoration was then placed in an ultrasonic cleaner filled with distilled water for 4 min [26]. Then it was silanated (Calibra silane) and heated using a furnace for 1 min [28], and universal adhesive was applied (One coat7) according to the manufacturer’s instructions as previously described.

### Outcome assessment

The marginal integrity of both nano-hybrid composite blocks (Brilliant Crios) and ceramic blocks (IPS e.max CAD was evaluated by mirror and explorer by two trained calibrated examiners (HS and MF) with more than ten years of clinical experience using modified USPHS criteria [17]. This was performed post-cementation and after 6, 12 and 24 months.

### Statistical analysis

Categorical and ordinal data were presented as frequencies and percentages. Categorical data were analyzed using the chi-square test. Ordinal data were analyzed using the Mann-Whitney U test for intergroup comparisons and Freidman’s test followed by the Nemenyi post hoc test for intragroup comparisons. Numerical data were presented as mean and standard deviation values. They were analyzed for normality using the Shapiro-Wilk test. Data were found to be normally distributed and were analyzed using the independent t-test. The significance level was set at *p* ≤ 0.05 within all tests. Statistical analysis was performed with R statistical analysis software version 4.1.3 for Windows (R Core Team (2022). R: A language and environment for statistical computing. R Foundation for Statistical Computing, Vienna, Austria. URL https://www.R-project.org/.)

## Results

**Fig. 1:**
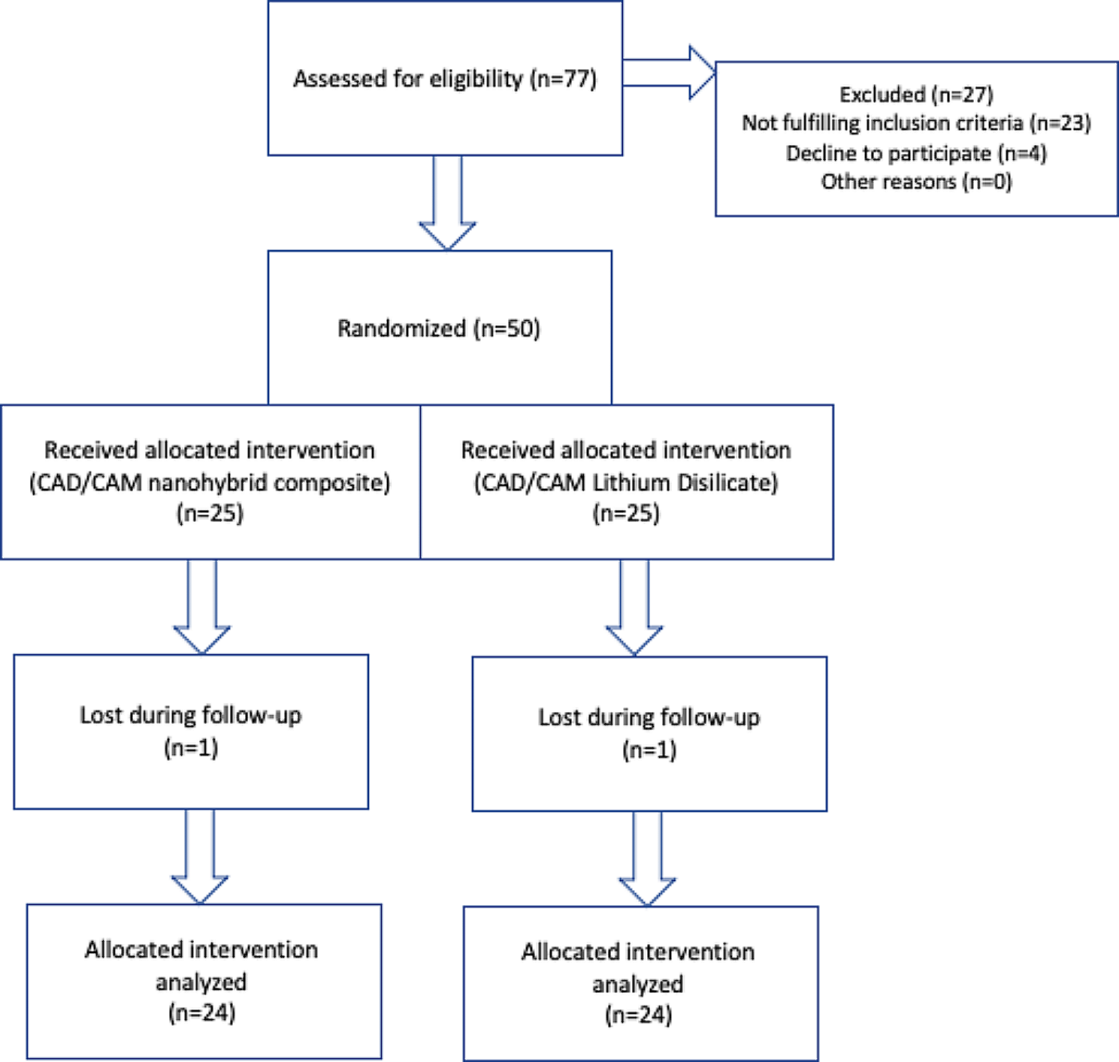
Participants Flow Diagram

Intergroup comparisons for demographic data showed no significant differences between both groups regarding sex (*p* = 1) and age (*p* = 0.582). Recruitment started 1/1/2021 and stopped 31/3/2021 after enrollment of target population. Follow-up started 1/1/2023 and was extended till 31/3/2023. All assigned participants received the allocated intervention. 48 participants completed the analysis and were analyzed for the outcomes. Two participants, one from each group, did not show-up during the follow-up period. Participants flow diagram is presented in Fig. 1.

The inter-evaluator reliability had a kappa value 0.98. Frequencies and percentages of outcome scores in both groups are presented in Tables (2–4).

**Table 2:** Frequencies and percentages of marginal integrity scores in both groups

Follow-up	Marginal integrity		Nano composite	Emax	***p***-value
**Baseline**	**Alpha-1**	**n**	25	25	**1ns**
		**%**	100.0%	100.0%	
	**Alpha-2**	**n**	0	0	
		**%**	0.0%	0.0%	
	**Alpha-3**	**n**	0	0	
		**%**	0.0%	0.0%	
	**Bravo 1**	**n**	0	0	
		**%**	0.0%	0.0%	
	**Bravo 2**	**n**	0	0	
		**%**	0.0%	0.0%	
	**Delta**	**n**	0	0	
		**%**	0.0%	0.0%	
	**Dropout**	**n**	0	0	
		**%**	0.0%	0.0%	
**6 months**	**Alpha-1**	**n**	25	20	0.021*
		**%**	100.0%	80.0%	
	**Alpha-2**	**n**	0	0	
		**%**	0.0%	0.0%	
	**Alpha-3**	**n**	0	5	
		**%**	0.0%	20.0%	
	**Bravo 1**	**n**	0	0	
		**%**	0.0%	0.0%	
	**Bravo 2**	**n**	0	0	
		**%**	0.0%	0.0%	
	**Delta**	**n**	0	0	
		**%**	0.0%	0.0%	
	**Dropout**	**n**	0	0	
		**%**	0.0%	0.0%	
**1 year**	**Alpha-1**	**n**	21	17	**0.150ns**
		**%**	84.0%	68.0%	
	**Alpha-2**	**n**	2	0	
		**%**	8.0%	0.0%	
	**Alpha-3**	**n**	0	0	
		**%**	0.0%	0.0%	
	**Bravo 1**	**n**	0	1	
		**%**	0.0%	4.0%	
	**Bravo 2**	**n**	0	3	
		**%**	0.0%	12.0%	
	**Delta**	**n**	0	1	
		**%**	0.0%	4.0%	
	**Dropout**	**n**	2	3	
		**%**	8.0%	12.0%	
**2 years**	**Alpha-1**	**n**	21	17	**0.150ns**
		**%**	84.0%	68.0%	
	**Alpha-2**	**n**	0	0	
		**%**	0.0%	0.0%	
	**Alpha-3**	**n**	2	0	
		**%**	8.0%	0.0%	
	**Bravo 1**	**n**	0	0	
		**%**	0.0%	0.0%	
	**Bravo 2**	**n**	0	4	
		**%**	0.0%	16.0%	
	**Delta**	**n**	0	1	
		**%**	0.0%	4.0%	
	**Dropout**	**n**	2	3	
		**%**	8.0%	12.0%	
***p*** **-value**			**1ns**	**1ns**	

Values with different superscript letters within the same vertical column are significantly different *; significant (*p* ≤ 0.05) ns; non-significant (*p* > 0.05)a

**Table 3:** Frequencies and percentages of marginal discoloration scores in both groups

Follow-up	Marginal discoloration		Nano composite	Emax	***p***-value
**Baseline**	**Alpha**	**n**	25	25	**1ns**
		**%**	100.0%	100.0%	
	**Bravo 1**	**n**	0	0	
		**%**	0.0%	0.0%	
	**Bravo 2**	**n**	0	0	
		**%**	0.0%	0.0%	
	**Charlie**	**n**	0	0	
		**%**	0.0%	0.0%	
	**Dropout**	**n**	0	0	
		**%**	0.0%	0.0%	
**6 months**	**Alpha**	**n**	25	20	0.021*
		**%**	100.0%	80.0%	
	**Bravo 1**	**n**	0	2	
		**%**	0.0%	8.0%	
	**Bravo 2**	**n**	0	3	
		**%**	0.0%	12.0%	
	**Charlie**	**n**	0	0	
		**%**	0.0%	0.0%	
	**Dropout**	**n**	0	0	
		**%**	0.0%	0.0%	
**1 year**	**Alpha**	**n**	21	17	**0.160ns**
		**%**	84.0%	68.0%	
	**Bravo 1**	**n**	2	1	
		**%**	8.0%	4.0%	
	**Bravo 2**	**n**	0	3	
		**%**	0.0%	12.0%	
	**Charlie**	**n**	0	1	
		**%**	0.0%	4.0%	
	**Dropout**	**n**	2	3	
		**%**	8.0%	12.0%	
**2 years**	**Alpha**	**n**	21	17	**0.194ns**
		**%**	84.0%	68.0%	
	**Bravo 1**	**n**	0	0	
		**%**	0.0%	0.0%	
	**Bravo 2**	**n**	2	4	
		**%**	8.0%	16.0%	
	**Charlie**	**n**	0	1	
		**%**	0.0%	4.0%	
	**Dropout**	**n**	2	3	
		**%**	8.0%	12.0%	
***p*** **-value**			**1ns**	**1ns**	

Values with different superscript letters within the same vertical column are significantly different *; significant (*p* ≤ 0.05) ns; non-significant (*p* > 0.05)

**Table 4:** Frequencies and percentages of restoration fracture scores in both groups

Follow-up	Restoration fracture		Nano composite	Emax	***p*** **-value**
**Baseline**	**Alpha**	**n**	25	25	**1ns**
		**%**	100.0%	100.0%	
	**Bravo**	**n**	0	0	
		**%**	0.0%	0.0%	
	**Charlie**	**n**	0	0	
		**%**	0.0%	0.0%	
	**Delta**	**n**	0	0	
		**%**	0.0%	0.0%	
	**Dropout**	**n**	0	0	
		**%**	0.0%	0.0%	
**6 months**	**Alpha**	**n**	25	25	**1ns**
		**%**	100.0%	100.0%	
	**Bravo**	**n**	0	0	
		**%**	0.0%	0.0%	
	**Charlie**	**n**	0	0	
		**%**	0.0%	0.0%	
	**Delta**	**n**	0	0	
		**%**	0.0%	0.0%	
	**Dropout**	**n**	0	0	
		**%**	0.0%	0.0%	
**1 year**	**Alpha**	**n**	23	20	**0.152ns**
		**%**	92.0%	80.0%	
	**Bravo**	**n**	0	0	
		**%**	0.0%	0.0%	
	**Charlie**	**n**	0	2	
		**%**	0.0%	8.0%	
	**Delta**	**n**	0	0	
		**%**	0.0%	0.0%	
	**Dropout**	**n**	2	3	
		**%**	8.0%	12.0%	
**2 years**	**Alpha**	**n**	22	20	**0.512ns**
		**%**	88.0%	80.0%	
	**Bravo**	**n**	1	0	
		**%**	4.0%	0.0%	
	**Charlie**	**n**	0	0	
		**%**	0.0%	0.0%	
	**Delta**	**n**	0	2	
		**%**	0.0%	8.0%	
	**Dropout**	**n**	2	3	
		**%**	8.0%	12.0%	
***p*** **-value**			**1ns**	**1ns**	

Values with different superscript letters within the same vertical column are significantly different *; significant (*p* ≤ 0.05) ns; non-significant (*p* > 0.05)

After six months, the inter-group comparison showed a significant difference in marginal integrity between both groups (*p* < 0.05), with all the cases (100%) in the nano-hybrid composite group having an alpha-1 score, while 80% (*n* = 20) of the cases in the Emax group had an alpha-1 score and 20% (*n* = 5) of them had an alpha-3 score. There was also a significant difference in marginal discolouration (*p* = 0.021), with all cases (100%) in the nano-hybrid composite group having an alpha-1 score. In comparison, in the Emax group, 80% (n=) of the cases (80%) had an alpha-1 score, while 8.0% (*n* = 2) of the cases had a bravo-1 score and 12.0% (*n* = 3) of them had a bravo-2 score.

At the 12- and 24-months observation points, there were no differences between the groups as regarding marginal integrity and discolouration (*p* > 0.05). Regarding the incidence of restoration fracture, there were no differences between either group at all observation points (*p* > 0.05).

The intra-group comparisons over time showed no differences in marginal integrity, marginal discolouration or incidence of fracture between nano-composite or Emax indirect restorations at all observation points (*p* = 1).

## Discussion

The purpose of this randomized clinical trial was to evaluate the longitudinal clinical performance of ceramic and composite chairside CAD/CAM partial posterior restorations over two years of clinical service.

Several factors contribute to the longevity of indirect restorations, including caries activity, occlusal load, and the clinician’s experience. Therefore, patients with high caries risk index and unusual occlusal habits were excluded from the study [29]. For both types of restoration, the cement gap space was set to 100 μm using Exocad software according to Sokolowski et al. [30] who stated that utilizing a cement layer less than 25 μm, resulted in high hygroscopic expansion stresses, and that exceeding 200 μm generated significant contraction stresses, and that a cement gap of 100 μm appears clinically acceptable. A dual-cure resin cement was used for cementation of the indirect restorations because it can compensate for the limited light transmission, hence enables complete polymerization in areas that are difficult for light penetration [31].

Three important clinical features, marginal integrity, marginal discolouration, and fracture incidence were selected for comparison and monitored over the observation period using the USPHS criteria which was refined to create descriptors with potentially finer discrimination to detect minor changes over time [14]. The modified USPHS criteria have been widely regarded as reliable and standard methods for evaluating the clinical performance of ceramic restorations in various published literature.

 [32, 33]. However, it is worth noting that several published studies have suggested that the modified USPHS criteria are less practical and less relevant, with limited sensitivity and categories that may not comprehensively reflect the clinical success of restorations compared to the FDI criteria [34, 35].

Regarding marginal integrity and marginal discoloration, the results of this study showed that initially, all the restorations in both groups had an alpha-1 score. This was predicted due to following all the common principles for indirect adhesive restorations regarding cavity preparation and restoration fabrication. Cavo-surface margin preparation and finishing into intact enamel, an important predictor of restoration survival [36], provided clear cavity margins for accurate scanning. Hence a precise design and fit of the final restoration were achieved, along with following all the literature recommendations regarding the bonding, cementation, finishing and polishing protocols for composite and ceramic restorations. All these factors collectively led to the perfect initial marginal integrity. After six months, a significant difference (*p* < 0.05) in marginal integrity and marginal discolouration existed between both groups in favour of the restorations milled from the nano-hybrid composite blocks. This is probably attributed to the nature of the materials used, where the ceramic blocks have a higher brittleness index (BI) in comparison to the nano-hybrid composite blocks, which renders them more susceptible to marginal chipping [37]. Also, a purported advantage of the nano**-**hybrid composites is that they wear at a similar rate to the resin cement, enabling them to maintain good marginal adaptation [14]. Moreover, degradation of the resin-based luting cement is more likely to occur under functional occlusal loading of the ceramic restorations, which have a lower modulus of elasticity than the tooth structure and the nano**-**hybrid composite material [38]. This agrees Archibald et al. [29] who reported cement wear with consequent marginal discoloration of ceramic indirect restorations at one-year follow-up. Conversely, in a laboratory study, Yildirim et al. [39] reported lower marginal adaptation for the indirect composite restoration (Lava Ultimate) and the hybrid ceramic restoration (Vita Enamic) than for the glass–ceramics (IPS e.max). Yildirim et al. [39] used a CEREC MC XL clinical-type milling unit with a 1.2-mm-diameter rotary instrument. However, smaller-diameter rotary instruments are recommended to capture finer curvature details and achieve more accurate results. Additionally, other variables, such as the virtual space configuration in the software, intrinsic properties of the CAD/CAM system, and speed of the rotary milling instruments, may also affect the outcomes [40].

After 12 and 24 months, there were no significant differences (*p* > 0.05) in the marginal integrity or marginal discolouration between both groups. This agrees Hassan et al. [40] who reported similar marginal adaptation for ceramic and composite indirect restorations after 2 years. Yet, disagrees Pallesen et al. [41] who reported a superiority for the indirect composite restorations after 11 years, attributed to the smooth interface between the indirect resin restoration and the resin cement, as they have similar mechanical properties.

Fracture of the restorative material has been reported as the main cause of failure in partial indirect restorations in posterior teeth and IPS e.max CAD [42, 43]. Regarding the incidence of restoration fracture in our study, there were no differences between either group at all observation points (*p* > 0.05). This can be attributed to the good mechanical properties of the used CAD/CAM blocks and the successful bonding of the restorations to the tooth structure. This improves stress distribution and response to the masticatory forces, preventing stress amplification in poorly bonded restorations [29]. Also, the use of the CAD/CAM technology enables control of the thickness of the luting resin cement, hence minimising the negative influence of polymerization shrinkage stresses [44, 45]. This agrees with previous published studies [46, 47]. However, it partially agrees Fasbinder et al. 2019 [14] who reported a higher fracture probability, yet not statistically significant, for Empress CAD onlays than the composite Lava Ultimate onlays after five years of clinical service, and Aslan et al. [48] who reported a minor fracture in a single case restored with a lithium disilicate restoration after one year of clinical follow-up, which also required a partial replacement.

Limitations of the present trial include a lack of standardization of the occlusal load as this is not a split-mouth design. However, patients with wear facets and parafunctional habits were excluded from participation. Also, a longer follow-up period is advised to evaluate the primary and secondary outcomes using modified USPHS criteria. Concerning the marginal integrity, it is recommended not to rely on clinical examination only, but also, it can be supported with the investigation of image analysis of scanned replicas.

In conclusion, the proposed null hypothesis should be accepted concerning the clinical performance after two years of recall as both CAD/CAM restorative materials evaluated exhibited a similar clinical performance after two years of service that need to be confirmed in long-term evaluations. This aligns with the results of previous studies [32, 46, 49, 50]. A recent systematic review also emphasized that indirect resin-based composite restorations are dependable materials for partial-coverage restorations, with clinical performance comparable to that of glass–ceramic restorations [15, 40].

Clinical relevance: The CAD/CAM nano-hybrid composite blocks are as reliable as Lithium disilicate for restoring mutilated vital teeth. However, clinicians must know the lack of knowledge regarding longer-term outcomes.

## Data Availability

Data are available from the corresponding author upon reasonable request and with permission of Cairo University - faculty of Dentistry.
